# Different effects of fluid loading with saline, gelatine, hydroxyethyl starch or albumin solutions on acid-base status in the critically ill

**DOI:** 10.1371/journal.pone.0174507

**Published:** 2017-04-05

**Authors:** Angélique M. E. Spoelstra–de Man, Annemieke Smorenberg, A. B. Johan Groeneveld

**Affiliations:** 1 Department of Intensive Care, Vrije Universiteit Medical Center, Amsterdam, The Netherlands; 2 Department of Internal Medicine, Ziekenhuis Amstelland, Amstelveen, The Netherlands; 3 Department of Intensive Care, Erasmus Medical Center, Rotterdam, The Netherlands; Medizinische Universitat Graz, AUSTRIA

## Abstract

**Introduction:**

Fluid administration in critically ill patients may affect acid-base balance. However, the effect of the fluid type used for resuscitation on acid-base balance remains controversial.

**Methods:**

We studied the effect of fluid resuscitation of normal saline and the colloids gelatine 4%, hydroxyethyl starch (HES) 6%, and albumin 5% on acid-base balance in 115 clinically hypovolemic critically ill patients during a 90 minute filling pressure-guided fluid challenge by a post-hoc analysis of a prospective randomized clinical trial.

**Results:**

About 1700 mL was infused per patient in the saline and 1500 mL in each of the colloid groups (**P**<0.001). Overall, fluid loading slightly decreased pH (P<0.001) and there was no intergroup difference. This mildly metabolic acidifying effect was caused by a small increase in chloride concentration and decrease in strong ion difference in the saline- and HES-, and an increase in (uncorrected) anion gap in gelatine- and albumin-loaded patients, independent of lactate concentrations.

**Conclusion:**

In clinically hypovolemic, critically ill patients, fluid resuscitation by only 1500–1700 mL of normal saline, gelatine, HES or albumin, resulted in a small decrease in pH, irrespective of the type of fluid used. Therefore, a progressive metabolic acidosis, even with increased anion gap, should not be erroneously attributed to insufficient fluid resuscitation.

**Trial registration:**

ISRCTN Registry ISRCTN19023197

## Introduction

**Fig 1:**
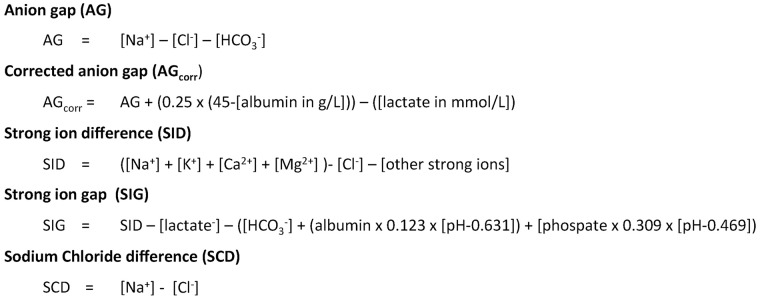
Commonly used formulas to assess acid base status according to Stewart and Henderson-Hasselbalch.

In shock, tissue hypoperfusion and concomitantly lactic acidosis can be treated with fluid administration. However, the type of fluid used for resuscitation in critically ill patients may also affect acid-base balance and can decrease blood pH. The mechanisms and short-term effects among the various fluids of this post infusion or dilution acidosis in clinical practise are largely unknown. This could lead to erroneous conclusions when blood pH is regarded as a marker of evalutating hypovolemia and tissue hypoxia [1]. The effects of fluid types on acid base status are evaluated using the anion gap according to Henderson-Hasselbalch and the strong ion difference (SID) according to Stewart (Fig 1) [2]. The anion gap is influenced by the change in (unmeasured) ions, and increases in the presence of a toxine, lactate, or ketoacids. The SID indicates the difference between strong cations and anions and is influenced by the total plasma concentration of weak non-volatile acids (Atot). It decreases with an increase in unmeasured anions and concomintant acidosis. The difference between sodium and chloride concentration has been shown to correlate good with the SID and corrected anion gap as a measure for the strong ion gap [3,4].

A post infusion acidosis can be the result of hyperchloraemia with decreased apparent strong ion difference or an increased anion (or strong ion) gap. [2,5,6]. The hyperchloraemic metabolic acidosis following infusion of relatively large volumes of normal saline, exceeding 2 L as studied in healthy volunteers, is well known and has a normal anion gap with a decreased strong ion difference. Colloids like albumine and gelatines are weak acids, whereas hydroxyethyl starch (HES) is not, and may also result in a metabolic acidosis with an increase in anion gap with a concomitant increase in Atot [2, 6–16]. There are only few studies comparing the effect of different fluids on acid-base balance in human subjects. Studies in healthy volunteers suggest a greater increase in chloride and decrease in pH after starch than equidosed albumin infusion. Also, there was a greater increase in chloride and decrease in bicarbonate with starch and saline infusion when compared to gelatine [17,18]. Studies in surgical patients did not show differences in decrease of blood pH comparing hypertonic saline or albumin versus HES [19,20]. However, these studies include small numbers of healthy volunteers or patients ranging from 10 to 40. The SAFE study included a larger number of critically ill patients, in which resuscitation with saline or albumin solution over mutiple days was compared. It showed a greater increase in chloride in the albumin group when large volumes were administered. The pH and bicarbonate increases did not differ amongst groups [21]. However, the amount of fluid administered varied and many factors may have affected the pH.

Following these controversies, we set out to study and compare the short term effects on acid-base balance and their mechanisms among currently used non-balanced resuscitation fluids, including saline, gelatine, HES and albumin solutions, in clinically hypovolaemic critically ill patients and subjected them to a 90-min standard fluid challenge protocol. The hypothesis was that these fluids differ in their short term effects on acid-base balance, when roughly similar volumes are infused.

## Patients and methods

This study was a post hoc analysis of a prospective, stratified, randomized, single blinded and single centre clinical trial concerning the effect of various fluid types in critically ill patients. We studied four groups of patients based upon fluid type given: with saline, gelatine, hydroxyethyl starch or albumin. The primary goal of this trial was to study the pulmonary and cardiac effects of crystalloid and colloid fluid loading in various patient subgroups. In succession, we included patients after cardiac and vascular surgery, with sepsis and other major surgery (S1 and S2 Text). The first part of the study encompassed the inclusion of 68 patients undergoing cardiac and major vascular surgery [22–25]. For the second stage 24 septic patients and 24 patients with major surgery were included [26–28]. For the current analysis, all patients were taken together and analysed based upon fluid type administered. Previous data has been published regarding the prediction of fluid responsiveness [24,25,28] and the cardiac and pulmonary effects of fluid loading in cardiac and major vascular surgery [22,23] and in septic and non-septic patients [24,25] when inclusion in one patient cohort after the other had been completed. This explains publications dated before the end of the complete study.

The study was approved by the Ethics Committee of the Vrije Universiteit Medical Centre (P2000.021 24-1-2000) and was performed in accordance with the 1964 Declaration of Helsinki. The study was registered prospectively at the central committee on research involving human subjects, the Netherlands. Written informed consent was obtained for all patients. Patients were enrolled from 8-8-2000 to 28-10-2003. At time of inclusion registration in a public database was not required. Research and human resources were made available belated for analysis and due to an increasing tendency to register studies to prevent publication bias, the study was retrospectively registered at the ISRCTN (ISRCTN19023197). Previous subanalysis have been published (all mentioned in the methods section and in the supporting information) without pretrial registration. There are no ongoing and related trials concerning this study protocol.

### Patients

Mechanically ventilated patients after cardiac and vascular surgery, with sepsis and other major surgery were included, who were clinically presumed hypovolaemia, defined as a pulmonary capillary wedge pressure (PCWP) below 10 mmHg in the presence of a pulmonary artery catheter and proper wedging or a central venous pressure (CVP) below 8 mmHg at positive end-expiratory pressure (PEEP) ≤15 cm H_2_O and below 12 mm Hg when PEEP >15 cm H_2_O in the presence of a central venous catheter, and a systolic arterial pressure <110 mmHg in the absence of vasopressor therapy. Inclusion criteria therefore also included the presence of a pulmonary artery or central venous catheter. Exclusion criteria were: age >78 years, pregnancy, known anaphylactoid reactions to colloids, and a preterminal setting with a life expectancy less than 24 h. Sepsis was defined by two or more of the following: abnormal body temperature (>38°C, <36°C), tachycardia (>90/min), tachypnoea (>20/min or partial pressure of carbon dioxide in arterial blood (PCO_2_) <32 mm Hg), abnormal white blood cell counts (<4, >12 x10^9^/L) and microbiologically proven or clinically evident source of infection. The origin of sepsis was defined by clinical signs and symptoms, imaging techniques, and positive local and/or blood cultures.

### Study protocol

**Fig 2:**
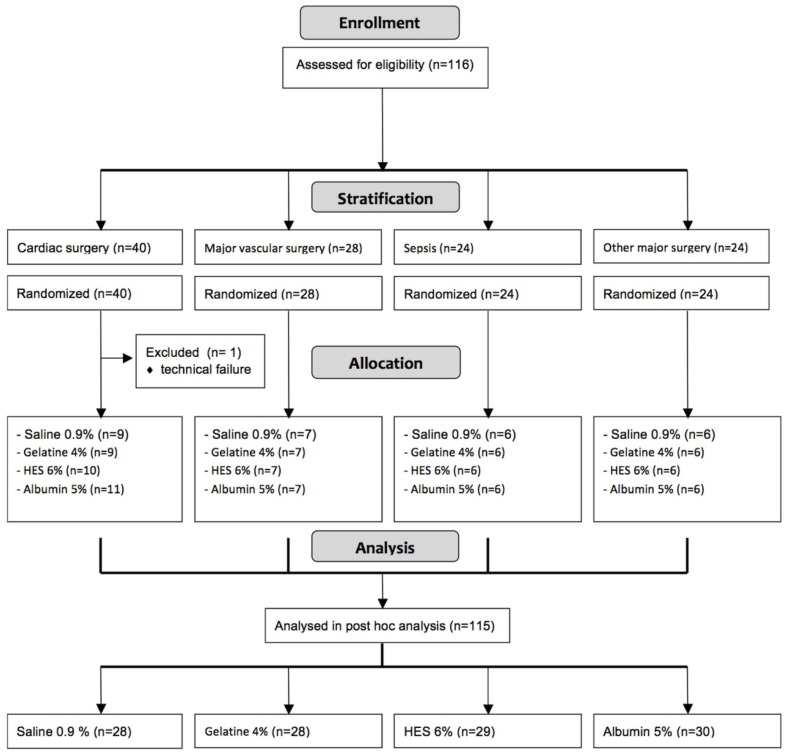
Patient population flow chart. N; number. HES; hydroxyethyl startch.

The hospital pharmacy assigned the patients randomly, via the sealed envelope method with an allocation ratio of 1:1:1:1, to the various fluid types (saline, gelatine 4%, HES 6% and albumin 5%) after stratification for cardiac surgery (n = 39), major vascular surgery (n = 28), other major surgery and trauma (n = 24) or sepsis (n = 24) (Fig 2). Within 3 hours after arrival of the patient in the intensive care unit (ICU), the study protocol was started. Demographic characteristics were recorded, including the acute physiology and chronic health evaluation (APACHE-II) score, and baseline (t = 0 min) measurements of haemodynamics were performed and blood samples taken. Fluids were dosed during 90 min on the basis of a response in predefined filling pressure changes, as measured by the pulmonary artery or, if absent, a central venous catheter according to the protocol as previously described [21,23]. The maximum amount of fluid infused was 1800 mL in the 90 min fluid loading protocol, targeting a CVP and PCWP of 13 and 15 mmHg, respectively. After fluid loading, measurement of haemodynamics and blood sampling were repeated and the study protocol was ended at t = 180 min. Concomitant infusion with vasopressor and sedative medication and ventilator settings remained unchanged during fluid loading. All care was given by physicians unaware of group assignment in the study protocol.

### Composition of study fluids

Normal saline (NaCl 0.9%) contains sodium 154 mmol/L and chloride 154 mmol/L (strong ion difference zero mEq/L). Gelofusine^R^ (B Braun Medical, Melsungen, Germany) contains succinylated gelatine 40 g/L, sodium 154 mmol/L and chloride 120 mmol/L (strong ion difference of 34 mEq/L). Hemohes^R^ (HES, B Braun Medical, Melsungen, Germany) consists of 60 g/L HES (200/0.45–0.55) dissolved in normal saline and albumin of 50 g/L albumin (Cealb^R^ 20%, Sanquin, the Netherlands) dissolved in normal saline.

### Measurements

The pH and PCO_2_ were measured by blood gas analysis (Rapid Lab 865, Bayer Diagnostics, Tarrytown, NY, USA, at 37°C). Actual bicarbonate and base excess values were calculated according to the recommendations of the National Committee for Clinical Laboratory standards. Serum sodium, chloride, albumin and lactate concentrations were measured using central chemical analyzers (Roche Modular P800 Module, Roche Diagnostics, Indianapolis, IN, USA). We calculated the relative plasma volume changes in percentage from (Hb0/Hb90)–((1-Hct90/1-Hct0)), in which Hb is haemoglobin and Hct haematocrit, measured at 0 min and 90 min (Sysmex SE-9000, Sysmex Corporation, Kobe, Japan) [29]. We calculated, in each patient, the sodium chloride concentration difference as a measure of apparent strong ion difference and the corrected anion gap as a measure of strong ion gap [3,4,28]. The anion gap (AG) was calculated from sodium minus chloride and bicarbonate concentrations and the corrected AG from AG + (0.25 x (45-[albumin in g/L]))–(lactate in mmol/L) to estimate unmeasured, non-lactic acid, anions. Hence, we integratively used physochemical (according to Stewart) and Henderson-Hasselbalch principles to assess acid base status [2,5].

### Statistical analysis

Data were prospectively collected for the current study over various patient subgroups, for which no separate power could be calculated a priori. Statistical analysis was performed with the help of SPSS 21 (IBM SPSS). Data were expressed as mean (standard deviation) since they were mostly normally distributed. A paired Student t test was used to evaluate changes in time for the whole group. A one-way analysis of variance followed by a Tukey’s test for post hoc comparisons was used to evaluate the differences in fluid effects. Generalized estimating equations (GEE) were used to judge the effect of fluid type on changes in study variables, adjusted for type of patient (cardiac surgery, major vasculair surgery, orther major surgery, or trauma). A two-tailed P<0.05 was accepted as the level of significance. Exact P values >0.001 are given.

## Results

116 patients were enrolled in the study. 1 patient was excluded due to technical failures and 115 patients were included in the final analysis. Patient characteristics are listed in Table 1. Groups were comparable. Fourteen patients died in the ICU (cardiac surgery: 1, vascular surgery: 1, sepsis: 9, trauma: 3). There were no signs of significant bleeding as blood drainage was < 50 mL/h in all patients. Plasma volume expansion differed between fluid types and was lower in saline compared to colloid fluid loading (P<0.001).

**Table 1:** Baseline characteristics and fluid administration.

	NaCl 0.9% n = 28	Gelatine 4% n = 28	HES 6% n = 29	Albumin 5% n = 30
Age, years	61 (12)	61 (13)	60 (13)	60 (9)
Sex, female	5 (18%)	3 (11%)	10 (34%)	8 (27%)
Weight, kg	78 (12)	82 (13)	75 (11)	79 (16)
Height, m	1.75 (0.08)	1.77 (0.07)	1.72 (0.09)	1.71 (0.20)
APACHE II	10 (5)	11 (5)	11 (4)	11 (4)
Fluid infused, mL	1723 (209)	1509 (328)	1441 (295)	1553 (258)
Hb, mmol/L,				
T = 0	6.2 (1.2)	5.7 (0.9)	5.7 (1.1)	5.9 (1.2)
T = 90*,**	6.1 (0.9)	5.0 (0.7)^A^	5.0 (0.8)^A^	5.2 (0.9)^A^
Change in PV, %**	5 [18–24]	18 [-8-49]^A^	21 [-11-50]^A^	16 [2–61]^A^

Values are mean (SD) or number (percentage), where appropriate. Abbreviations: PV, plasma volume; HES, hydroxyethyl starch.

* P<0.001 for decrease in whole group;

** P<0.001 between fluids;

^A^ P<0.001 for change vs saline.

### Acid-base balance and electrolytes

**Fig 3:**
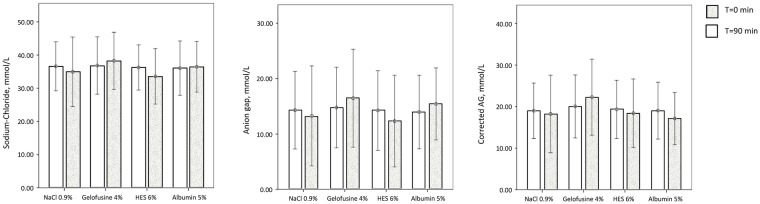
Sodium-chloride differences and (un)corrected anion gaps in various fluid types. (A) Mean (error bars represent +/- standard deviation) of sodium-chloride differences (Na^+^-Cl^-^) at baseline and after 90 minutes loading of various fluid types. For sodium-chloride difference: P = 0.032 T = 90 vs T = 0, P<0.001 for change between groups and P<0.001 for change in gelatine or albumin vs saline and hydroxyethyl starch (HES). (B) Mean of uncorrected anion gaps at baseline and after 90 minutes loading of various fluid types. P<0.001 for change between groups, P = 0.027 or lower for change in gelatine vs saline and hydroxyethyl starch (HES) and change in albumin vs saline and HES. P<0.001 for change in HES vs gelatine and albumin. (**C)** Mean of corrected anion gaps (AG) at baseline and after 90 minutes loading of various fluid types. P<0.001 for change between groups, P<0.001 for change in gelatine vs saline, hydroxyethyl starch (HES), and albumin.

There were no baseline differences. Overall, fluid loading decreased pH, bicarbonate and base excess. There were no intergroup differences, in spite of differences in intravascular volume expansion (P<0.001, Table 2). Overall, the PCO_2_ and lactate remained within normal limits and chloride concentrations increased and albumin decreased. Lactate decreased somewhat more in the albumin than gelatine group. Chloride increased in the saline and HES groups and was unchanged in the gelatine and albumin groups. The sodium chloride difference decreased in the saline and HES and hardly changed in the gelatine and albumin groups (Fig 3A). Albumin concentration decreased in the normal saline, gelatine and HES group, whereas it increased in the albumin group. Both corrected and uncorrected anion gap decreased overall. However, the uncorrected anion gap increased in the gelatine and albumin groups as compared to the decrease in the saline and HES groups (Fig 3B). The corrected anion gap increased in the gelatine group only (Fig 3C).

**Table 2:** Acid-base balance and electrolytes.

	NaCl 0.9% n = 28	Gelatine 4% n = 28	HES 6% n = 29	Albumin 5% n = 30
Sodium, mmol/L				
T = 0	142.5 (4.6)	144.8 (3.9)	144.4 (3.9)	143.4 (3.9)
T = 90	143.3 (4.6)	146.2 (3.7)	143.2 (3.7)	143.9 (3.9)
Chloride, mmol/L				
T = 0	105.9 (5.8)	107.7 (4.3)	108.2 (4.2)	107.2 (5.2)
T = 90*,**	108.3 (6.7)	107.7 (4.2)^A^	109.7 (3.8)	107.4 (4.9)^A^
Lactate, mmol/L				
T = 0	1.2 (0.6–4.2)	1.2 (0.6–3.7)	1.2 (0.5–3.7)	1.4 (0.5–3.4)
T = 90**	1.2 (0.7–4.1)	1.3 (0.6–3.6)	1.2 (0.5–3.1)	1.2 (0.5–2.9)^B^
Albumin, g/L				
T = 0	20.0 (5.1)	18.1 (4.1)	19.2 (3.5)	17.8 (6.8)
T = 90*,**	19.2 (5.3)	15.9 (3.7)^C^	15.0 (2.5)^C^	30.8 (5.3)^D^
pH				
T = 0	7.39 (0.06)	7.40 (0.07)	7.40 (0.07)	7.38 (0.06)
T = 90*	7.37 (0.06)	7.39 (0.06)	7.39 (0.06)	7.36 (0.06)
PCO_2_, mmHg				
T = 0	37.6 (5.5)	36.1 (5.5)	36.5 (8.6)	37.8 (5.6)
T = 90	37.9 (6.4)	37.4 (4.7)	36.5 (8.6)	37.8 (5.0)
Bicarbonate, mmol/L				
T = 0	22.3 (3.0)	22.1 (2.9)	22.0 (3.2)	22.2 (2.5)
T = 90*	21.6 (2.8)	22.1 (2.6)	21.5 (3.1)	21.3 (2.5)
Base excess, mmol/L				
T = 0	-2.0 (3.2)	-1.9 (3.3)	-2.0 (3.2)	-2.2 (3.0)
T = 90*	-3.0 (2.9)	-2.1 (3.2)	-2.6 (3.2)	-3.3 (3.0)
Corrected anion gap, mmol/L				
T = 0	19.0 (3.3)	20.0 (3.8)	19.3 (3.5)	19.0 (3.4)
T = 90**	18.2 (4.7)	22.3 (4.6)^E^	18.4 (4.0)	17.0 (3.2)

Values are mean (SD). Abbreviations: HES; hydroxyethyl starch;

* P = 0.002 or lower for whole group at t = 90 vs t = 0 min;

** P = 0.010 or lower for change between groups;

^A^ P = 0.004 or lower for change vs saline,

^B^ P = 0.005 for change vs gelatine,

^C^ P = 0.020 or lower for change vs saline and albumin,

^D^ P<0.001 for change vs other groups,

^E^ P = 0.007 or lower for change vs other groups.

### Multivariable analysis

The change in pH was independent of fluid and patient type, whereas the change in chloride, sodium-chloride difference and uncorrected or corrected anion gap depended on fluid type (P<0.001) and not on patient type (P = 0.14 or higher).

## Discussion

In this study in clinically hypovolaemic, critically ill patients, fluid loading of about 1.5–1.7 L only had a mildly metabolic acidifying effect, irrespective whether normal saline, gelatine, HES or albumin was used. This can be attributed to a small increase in chloride concentration in saline- and HES-, and in corrected anion gap in gelatine- and uncorrected anion gap in albumin-loaded patients, independent of lactate concentrations.

Although the amount of fluid used was higher in the normal saline group as compared to the colloid groups, it did not lead to a larger decrease of pH. This implies that there is no preference for type with regard to acid-base balance, among the investigated fluids, even though artificial colloids may be associated with other adverse effects compared to saline [30]. The intrinsic acidifying effect of these fluids, however, must always be considered as a possible cause of progressive metabolic acidosis. Indeed, normal saline and HES administration increased the chloride concentration, whereas chloride did not change in the gelatine and albumin groups.

The increase in chloride in the saline group is in accordance with the literature and our study suggests that massive amounts (>15 mL/kg/h for hours) are not required for the phenomenon to develop, as suggested previously [7, 9–16]. The albumin concentration increased in the albumin group but decreased in the other groups. Succinylated gelatine and albumin are weak acids, whereas HES is not [2,6,8]. Hence, the decrease in pH in the normal saline and HES group seems to be mainly caused by increased chloraemia and a decreased strong ion difference, and in the gelatine and albumin groups by an increase in weak acids elevating the anion gap, unless corrected for albumin concentrations in the albumin group. The acidifying mechanisms thus differ among these fluids. Hydroxyethyl starch and albumin are dissolved in normal saline, however, fluid infusion did not change plasma chloride concentration, what may be the result of a smaller amount of fluid infused. The effects described in this study on critically ill patients can also be explained by the equilibration of extracellular fluid with infused fluids and their respective strong ion differences and total concentration of weak acids (A_tot_) [2,6]. Normal saline and starches have reduced (zero) strong ion difference, whereas gelatine and albumin probably have increased A_tot_, as compared to plasma [2,6]. Hence, infusion of saline and starch may have resulted in a decreased strong ion difference and infusion of gelatine and albumin in an increased (uncorrected) anion gap and A_tot_. Our study suggests that massive amounts of fluids are not necessary for these dilutional effects.

In a sub-study of the large randomized controlled SAFE trial, albumin and saline fluid resuscitation similarly increased blood pH in the critically ill, but large-volume albumin resuscitation (>3 L in the first day) resulted in a greater increase in chloride [21]. The sustained effects of fluid resuscitation on acid-base balance were investigated over days and the results are therefore likely to be influenced by time and the progression or resolution of the underlying illness or other treatments. Our study shows that the acidifying short term effects of the different fluids on acid-base balance, which are less likely to be influenced by other factors, are opposite to that in the SAFE trial. The results of our study are also only partly in accordance with other trials investigating the effect of fluids on acid-base balance in non-critically ill patients [19,20]. In two studies on surgical patients, no differences in degree of acidosis were shown for (saline-dissolved) HES vs albumin or hypertonic saline [19,20]. One study comparing HES and albumin infusion showed an increased anion gap with albumin but a similar elevation of chloride in both groups [19]. Our results partly agree with the volunteer studies suggesting greater metabolic acidifying effect by elevating chloride of hetastarch than of albumin and greater rise in chloride with saline or HES than gelatine solutions [17,18].

Potassium (and other cation) concentrations were not available for full calculation of the strong ion difference and anion gap and this can be regarded as a limitation of the study. Nevertheless, since the concentration of potassium in the blood is relatively small as compared to sodium, bicarbonate and chloride, it is common practice to omit this variable [4,31]. Furthermore, a potential dilutional decrease in potassium concentration following fluid therapy would result in underestimations of the decreases in the anion gap we found in the saline and HES groups, unless offset by an acidosis-induced increase in potassium. Hence, our conclusions regarding fluid differences, using clinically amenable estimations of strong ion difference and anion gap [4,31], remain valid. We cannot exclude that the differences in volumes infused and plasma volumes expanded compounded our results and that the effect of saline was underestimated, even if equilibration of fluids and eletrolytes between plasma and interstitial fluids was similarly rapid among fluid types [21]. We did not administer fixed doses of fluids and dosed on the basis of changes in filling pressures for safety reasons [22,27]. Our study carries the advantage of comparing still commonly used non-balanced resuscitation fluids. We cannot decide, however, on potentially detrimental effects in the patients we studied. Finally, the relatively short study period and, on average, normal lactate levels, increases the likelihood that we studied changes evoked by the fluids themselves, rather than changes in patients metabolism. In our study, the physicochemical approach (strong ion difference) was clearly of adjunctive value to the Henderson-Hasselbalch approach in assessing the effects of fluids on acid-base balance, since the bicarbonate and base excess levels similarly declined among the resuscitation fluids (at constant PCO_2_).

## Conclusions

In our study of clinically hypovolemic, critically ill patients, fluid resuscitation by only 1.5–1.7 L of normal saline, gelatine, HES or albumin, resulted in a small decrease in pH, irrespective of the type of fluid used, due to a small increase in chloride concentration in saline- and HES-, and in (uncorrected) anion gap in gelatine- and albumin-loaded patients. The latter was independent of lactate concentrations. Therefore, progressive metabolic acidosis should not be simply attributed to inadequate fluid resuscitation, even with an increased anion gap and application of relatively small volumes.

## Supporting information

S1 Text Study overview including previously published data. (DOCX)

S2 Text Original study protocol. (DOC)

S1 Table Consort checklist. (DOC)
