# Supplementary material for: Different effects of fluid loading with saline, gelatine, hydroxyethyl starch or albumin solutions on acid-base status in the critically ill
Source: PLoS One. 2017 Apr 5;12(4):e0174507. doi: 10.1371/journal.pone.0174507 (PMC5381890; doi:10.1371/journal.pone.0174507)
Supplement: S1 Text — (DOCX) [file pone.0174507.s001.docx]

**Supporting information 1 Text: Overview of previous published data**

**Trial**

90 minutes filling pressure based fluid protocol after randomization for NaCl 0.9%, Gelofusin 4%, HES 5%, or albumin 5%.

**Goal:** To study the pulmonary and cardiac effects and effects on acid-base balance of crystalloid and colloid fluid loading, in various patient subgroups including patients after cardiac and vascular surgery, and patients with sepsis and other major surgery (Figure 1, manuscript).

**Patients**

*Inclusion criteria:* clinically presumed hypovolaemia, defined as a pulmonary capillary wedge pressure (PCWP) below 10 mmHg in the presence of a pulmonary artery catheter and proper wedging or a central venous pressure (CVP) below 8 mmHg at positive end-expiratory pressure (PEEP) ≤15 cm H_2_O and below 12 mm Hg when PEEP >15 cm H_2_O in the presence of a central venous catheter, and a systolic arterial pressure <110 mmHg in the absence of vasopressor therapy. Inclusion criteria therefore also included the presence of a pulmonary artery or central venous catheter.

*Exclusion criteria:* age >78 years, pregnancy, known anaphylactoid reactions to colloids, and a preterminal setting with a life expectancy less than 24 h.

**Study protocol**

The study protocol was started within 3 h after arrival. Patient characteristics were recorded, pulmonary and systemic haemodynamic variables were measured, and blood samples were taken. An anteroposterior chest radiograph was taken. A lung injury score was calculated based on the level of PEEP, the arterial  PO_2_/FI_O2_ ratio, total respiratory compliance and the number of quadrants with alveolar consolidations on the chest radiograph. The score ranges from 0 to 4, with values <2.5 denoting ALI and >2.5 ARDS. For the measurement of cardiac output (CO), intrathoracic blood volume (ITBV) and EVLW, the transpulmonary thermal/dye dilution technique was used. Pulmonary capillary permeability was measured by pulmonary leak index (PLI) for Gallium- 67 (67Ga)-labeled transferrin.

The hospital pharmacy assigned the patients randomly, via the sealed envelope method with an allocation ratio of 1:1:1:1, to various fluid types after stratification for cardiac surgery, major vascular surgery, other major surgery and trauma or sepsis. Patients had been assigned to NaCl 0.9%, GelofusineR (gelatin 40 g litre−1, B. Braun Melsungen AG, Germany, in 154/120 mmol litre−1NaCl), HES 6% (MW 200,000, substitution 0.45–0.55, HemohesR, B. Braun Melsungen AG, Germany, in saline) or albumin 5% (100 ml Cealb 20%, Sanquin, CLB, Amsterdam, The Netherlands, diluted in 300 ml of saline). After baseline measurements, fluids were given during 90 min on the basis of the response within predefined pressure limits (Table 1 manuscript), on the basis of the PCWP after appropriate wedging or the central venous pressure. The measurements including blood samples were repeated immediately after completing the fluid challenge (t=90 min). Diuresis was recorded.

**Part 1**

*Date:* 2000-2006
 Start patient inclusion: 8-8-2000
 End patient inclusion: 21-1-2003

*Patients:*N=68

- cardiac surgery n=40

- major vascular surgery n=28

*Exclusion:* n=1 due to technical failures

*Included:* n=67

**1.1 Goal:** To study the effects on volume expansion and myocardial function of colloids or

crystalloids in the treatment of hypovolaemic hypotension after cardiac and major vascular surgery [1].

**Hypothesis:** Colloid fluid loading, by maintaining plasma COP, results in a greater plasma volume expansion and cardiac output elevation with time than does saline fluid loading, after cardiac or vascular surgery and exogenous colloids would perform similarly to albumin in this respect

**Study type:** prospective, stratified, randomized, single blinded and single centre clinical trial

**Patients included in analysis:** n=67
**Publication:** Intensive Care Medicine **Publication year:** 2006

**1.2 Goal:** To study the effects on pulmonary function and lung oedema formation of colloids or

crystalloids in the treatment of hypovolaemic hypotension after cardiac and major vascular surgery [2].

**Hypothesis:** Colloid fluid loading would aggravate less oedema formation in the lungs than saline loading in the treatment of presumed hypovolaemia after major surgery, even if complicated by increased pulmonary permeability.

**Study type:** prospective, stratified, randomized, single blinded and single centre clinical trial

**Included in analysis:** n=67

**Publication:** British Journal of Anaesthesia **Publication date:** 2006

**1.3 Goal:** To study the ability of filling pressures and volumes to predict fluid responsiveness after cardiac or vascular surgery in normal and reduced systolic cardiac function [3].

**Hypothesis:** During fluid loading in patients with reduced systolic cardiac function as compared to those with normal function, filling pressures may be superior to filling volumes for predicting and monitoring of fluid responsiveness.

**Subgroup:** Only patients with completion of fluid loading with colloids and measurements up to t=90.
**Study type:** sub-study of a prospective randomized clinical trial

**Patients included in analysis:** n=32

**Publication:** Critical Care **Publication date:** 2011

**1.4 Goal:** To study the value of mixed venous O2 saturation and fluid responsiveness after cardiac or major vascular surgery [4].

**Hypothesis:** The value of SvO2 as a predictor and monitor of fluid responsiveness in critically ill and hypovolemic patients after cardiac or major vascular surgery depends on cardiac functions.

**Study type:** sub-study of a prospective randomized clinical trial

**Subgroup:** Only patients with completed fluid loading with colloids at t=90 and pulmonary artery derived measurements up to t=90.

**Patients included in analysis:** n=27

**Publication:** Journal of Cardiothoracic Surgery **Publication year:** 2013

**Part 2**

*Date:* 2000-2009
 Start patient inclusion: 13-12-2000
 End patient inclusion: 28-10-2003

*Patients:* 48 patients

- septic patients n=24

- other major surgery n=24

*Included:*n=48

**2.1 Goal:** To study the pulmonary effects of crystalloid and colloid fluid loading in septic and non-septic patients [5].

**Hypothesis:** Fluid loading with crystalloids compared with colloids gives more pulmonary edema formation in clinically hypovolemic patients and the difference between crystalloids and colloids in influencing edema formation decreases when permeability is increased, but the propensity for edema formation increases.

**Study type:** prospective, stratified, randomized, single blinded and single centre clinical trial

**Patients included in analysis:** n=48
 **Publication**: Critical Care Medicine

**Publication year**: 2009

**2.2 Goal:** To study the effect of crystalloid and colloid fluid leading on preload-recruitable cardiac output and stroke work in septic and non-septic patients [6].

**Hypothesis:** Because of differences in cardiac and vascular function, the hypothesis was that fluid loading with colloids result in a greater increase in cardiac output, more so in non-septic patients compared to septic patients.

**Study type:** prospective, stratified, randomized, single blinded and single centre clinical trial

**Patients included in analysis:** n=48

**Publication**: Intensive Care Medicine

**Publication year**: 2010

**2.3 Goal:** To evaluate and compare filling volumes to pressures in determining the cardiac response tof luid loading according to systolic cardiac function in sepsis-induced hypotension [7].

**Hypothesis:** cardiac dilatation is required to increase cardiac output upon fluid loading, even in dysfunctioning hearts.

**Study type:** sub-study of a prospective randomized clinical trial

**Subgroup:** Only patients receiving colloid fluid loading with both monitoring by central venous pressure and the transpulmonary dilution technique included into analysis.

**Patients included in analysis:** n=16

**Publication**: BMC Anesthesiology

**Publication year**: 2013

**Part 3**

*Date:* 2014-2016

*Patients:*N=116

- cardiac surgery n=40

- major vascular surgery n=28

- septic patients n=24

- other major surgery n=24

*Exclusion:* n=1 due to protocol error

*Included:* n=115

**3.1 Goal:** To study and compare the short term effects on acid-base balance and their mechanisms among currently used non-balanced resuscitation fluids, including saline, gelatine, HES and albumin solutions, in critically ill patients considered clinically hypovolaemic [current data].

**Hypothesis:** These fluids differ in their short term effects on acid-base balance, when roughly similar volumes are infused.

**Study type:** post-hoc analysis

**References**

1. Verheij J, van Lingen A, Beishuizen A, Christiaans HM, de Jong JR, Girbes AR, et al. Cardiac response is greater for colloid than saline fluid loading after cardiac or vascular surgery. Intensive Care Med. 2006: 32: 1030-8

2. Verheij J, van Lingen A, Rijnsburger ER, Veerman DP, Wisselink W, Girbes AR, et al. Effect of fluid loading with saline or colloids on pulmonary permeability, oedema and lung injury score after cardiac and major vascular surgery. Br J Anaesth. 2006: 96: 21-30

3. Trof RJ, Danad I, Reilingh MW, Breukers RM, Groeneveld AB. Cardiac filling volumes

versus pressures for predicting fluid responsiveness after cardiovascular surgery: the role of systolic cardiac function. Crit Care. 2011;15 :R73.

4. Kuiper AN, Trof RJ, Groeneveld AB. Mixed venous O2 saturation and fluid responsiveness after cardiac or major vascular surgery. J Cardiothorac Surg. 2013: 22: 8: 189

5. van der Heijden M, Verheij J, van Nieuw Amerongen GP, Groeneveld AB. Crystalloid or colloid fluid loading and pulmonary permeability, edema, and injury in septic and nonseptic critically ill patients with hypovolemia. Crit Care Med. 2009: 37: 1275-81

6 Trof RJ, Sukul SP, Twisk JW, Girbes AR, Groeneveld AB. Greater cardiac response of colloid than saline fluid loading in septic and non-septic critically ill patients with clinical hypovolaemia. Intensive Care Med. 2010; 36:697-701.

7. Trof RJ, Danad I, Groeneveld AJ. Global end-diastolic volume increases to maintain fluid responsiveness in sepsis-induced systolic dysfunction. BMC Anesthesiol. 2013; 22;13:12.
