# Supplementary material for: Different effects of fluid loading with saline, gelatine, hydroxyethyl starch or albumin solutions on acid-base status in the critically ill
Source: PLoS One. 2017 Apr 5;12(4):e0174507. doi: 10.1371/journal.pone.0174507 (PMC5381890; doi:10.1371/journal.pone.0174507)
Supplement: S2 Text — (DOC) [file pone.0174507.s002.doc]

**Supporting information 2 Text: study protocol**

**The effect of resuscitation fluids on micro­vascular fluid and protein extravasation and respira­tory variables in patients with or at risk for the acute respi­ra­tory distress syndrome (ARDS).**

Principal investigator:

Dr ABJ Groeneveld, internist-intensivist

Medical Intensive Care Unit

Free University Hospital

De Boelelaan 1117

1081 HV Amsterdam

The Netherlands

Version: definite, 2000

**Contents.**

page

Contents 2

Summary 3

Introduction 4

Study rationale, objectives and variables 4

Study design 9

Selection of patients 9

Material and methods; protocol 10

Analysis 11

Documentation and safety monitoring 12

Patients inconvenience 14

Ethical considerations and informed consent 14

References 15

Appendices I 19

II 22

III 24

**Summary.**

Major problems in critically ill patients in the intensi­ve care unit include circulatory and respiratory in­sufficiency. In treating circulatory insufficiency (hypovolemia), many patients receive various types of soluti­ons, but many of these solutions have (potential) adverse effects. The ideal resus­citation fluid should optimize the systemic circula­tion wit­hout adverse effects in the pulmo­nary circula­tion, and, in this respect, starch solutions are hypothesized to be better than other colloids by virtue of their specific sealing effects, and colloids are believed to be better than crystalloids by virtue of their colloid osmotic pressure and thereby fluid filtration inhibiting ef­fects, although hard evidence for these theoretical advanta­ges are still lacking. In fact, the precise effects of infusion of various types of flu­ids, eg crystalloid and colloid, including starch solutions, on lung protein and fluid balance and respiratory function during resuscita­tion from hypovole­mia, are largely unknown and may, among others, depend on the presence and degree of microvascular injury and increased permeability in the lung. This current research proposal thus aims at clarifying the efficacy of various solutions relative to the pulmo­nary side effects, in critically ill patients with hypovolemia, either complicated by a pulmonary microvascular injury and increased permeability, as manifested by the acute respira­tory distress syndrome (ARDS), or in patients at risk for ARDS (following 4 major etiologies), since the effect of the plasma colloid osmotic pressure would be lower, and that of sealing greater, in inhibiting protein and fluid filtration across the pulmonary microvasculature associated with impaired lung mechanics and gas exchange, in the former than in the latter condition, respectively.

**Introduction.**

During resuscitation from hypovolemia, a common problem in critical­ly ill patients and associated with a variety of underlying disorders, various infusion fluids can be used (1-3). The best resuscitation fluid is unknown, however, and clinicians are guided by various factors in their choice among fluids. These factors include the costs, ease of use (availability) and presence of expertise and effectiveness in terms of plasma volume expansion and increased cardiac output and oxygen delivery, versus associ­ated effects on the plasma colloid osmotic pressure, edema formation, and resultant potential pulmonary side effects. Indeed, a major thread of these solutions is fluid overload and formation of pulmonary edema, but, in this respect, fluids may differ in their associated risk for pulmonary complications during intravenous loading. Taken together, resuscita­tion fluids may compromise lung function, even though they may benefit other derangements of the critically ill patient. This is an important notion since many critically ill patients develop respiratory insufficiency on the basis of fluid overload, particularly in the course of acute lung injury and the acute respiratory distress syndrome (ARDS), associated with microvascular injury, increased permeability and non-cardiogenic pulmonary edema. The need for intubation and artifi­cal ventilation may be an impor­tant contri­bu­tory factor to death in patients with severe underlying illness (4).

**Study rationale, objectives and variables.**

Du­ring resuscitation from various causes of severe hypovolemia (and shock), the first step is to correct the volume deficit by infu­sing crystalloid or colloid fluids (1). The aim is to restore filling pressure of the heart and thereby cardiac output, arterial blood pressure and diure­sis. The pulmonary vascular pressures are increased during this resuscita­tion phase as well, so that fluid resuscitation may lead to pulmo­nary edema and impaired gas exchange and need for intubation and ventila­tion, particu­larly when the microvascular membrane is already damaged and leaky, associated with the prior shock episo­de and development of acute lung injury (1,5). This is a potentially harmful effect of fluid resusci­ta­tion and fluid types have been compared in the past regar­ding, among others, this detrime­ntal side effect in the presumption that colloid fluids that maintain plasma colloid osmotic pressure would, for a given increase in systemic and thus pulmonary pressu­res, result in less propen­sity for development of pulmonary edema than crystal­loid fluids that would, by virtue of dilution, decrease plasma colloid osmotic presssure (2,5). This effect would particularly become important in case of a moderate increase in microvascular permeability, since this would increase the propensity for edema formation (versus the normal situation). In fact, about 60% of patients at risk for ARDS in the course of severe sepsis, trauma, cardiac and vascular disease, have increased pulmonary microvascular permeability and discrete respiratory abnormalities, without fulfilling the full blown syndrome, which can be regarded as the "tip of the iceberg" of microvascular damage during acute lung injury (6). The risk for ARDS development after cardiopulmonary bypass surgery, major vascular surgery, multiple trauma and sepsis amounts to about 30 % (6-10). On the other hand, the importance of the plasma colloid osmotic pressure effect in inhibiting the formation of pulmonary edema decreases if the permeability increase is severe, as occurs during ARDS (9,11,12).

These physiologi­cally sound ideas have never been confirmed by the re­sults of clini­cal studies comparing the effects of crystalloids and colloids in resusci­tation from hypovolemia (1-3). In fact, recent meta-analyses have suggested a potential detrimental effect of colloids versus crystalloids, but this may have been caused, among others, by overdosing. In any case, it is general­ly accepted that less amounts of colloid soluti­ons are necessary for intrava­scular volume expansion than crystalloid fluids, because of less extrava­sation of colloids and thus fluid, but the use of albumin for this purpose has been discouraged mainly because of its associated costs and (the never confirmed) potential to transmit disease (13,14). Hence, colloid rather than crystal­loid fluid resuscitation may protect against organ edema and injury in various shock states (5,15,16). Arguments disfavo­ring colloids include lack of clear proof of diminis­hed pulmonary fluid overload and impaired gas exchange as compared to crystal­loid resuscitation, and their potential side effects. However, some surrogate markers of pulmonary edema such as oxygenation variables have been used to assess the effect of crystalloid and colloid fluids on lung fluid balance. Direct measurements of extravascular lung water (EVLW) as an endpoint for fluid resuscitation are scarce (4,17,18). Neverthe­less, colloid soluti­ons are widely used (and often preferred) for resusci­tation of hypovolemic patients and various types have been advoca­ted (1-3,17-19,21).

It remains con­trover­sial whether some colloids are superior to others, in the treatment of severe hypovo­le­mia (3). Artifi­ci­al colloid solutions include dextra­ns, gelatins and starch deri­vates (1,2,14). However, colloids may differ (2,19) in their effect on restora­tion of circulation and tendency for evoking pulmonary edema, even indepen­dently of mean molecular weight (MW) and associated colloid osmotic pressure. Some of the starch solutions (particularly those with relatively low MW of about 200.000 with less dispersion as opposed to the classical 450.00 MW hydroxye­thyl starches) may have additional bene­fits, as related to the presumed potenti­al to "seal capilla­ries" and to ameliorate increased perme­ability, as may clinically occur in patients with ARDS (2,9,20,21). This may both relate to a functional blockade of endothelial pores but also to changes in the microcirculation via beneficial interference with (expression of endothe­lial) clotting factors and leukocyte adhesion molecules (22,23). The ability of starch solutions to decrease enhanced permeabi­li­ty has been demon­strated in various animal models, including brain damage and ischemi­a/re­perfusion models (16,20,21,24). It remains to be proven, however, whether this is caused, as has been sugge­sted by others, by the intrinsic proper­ties of the compound, rather than by raising plasma colloid osmotic pressure (2,15,19-21,24). Nevertheless, pentastarch and hetas­tarch (with averaged MW higher than of albumin) resusci­tation during acute lung injury in animals has been shown to result in less transvascular (lymphatic) and transepi­thelial fluid transpor­t into alveoli, respectively, than resusci­tation with plasma (15,24). Otherwise, plasma albumin may, by interac­ting with endothe­lium, also decrease endothelial permeabili­ty in the microvas­culature of the lungs, thereby limiting transendo­thelial protein transport for a given pressure gradient and fluid transport (5,25).

Taken together, the current hypothesis is that starch solutions are superior to other colloids in preventing pulmona­ry complicati­ons (less protein and fluid extravasation, less deterioration of chest radio­graph and ventilatory variables), in patients with ARDS, and that the latter (albumin and gelatins) are superior to saline, during resuscitation from hypovole­mia in patients at risk for ARDS. The underlying hypothesis is that during ARDS, the effect of the plasma colloid osmotic pressure would be lower, and that of sealing greater, in inhibiting protein and fluid filtration across the pulmonary microvasculature associated with impaired lung mechanics and gas exchange, than in patients at risk for ARDS. In the current research proposal we will therefore compare, in a 2 hour period, fluid challenges with crystal­loids (normal saline), and the colloids albumin, gelatin (Gelofusine^R^, B.Braun Melsungen) and s­tarch (Hemohes^R^, B.Braun Melsungen) (at roughly equal colloid osmotic pressures) in their effects on blood chemis­try (hematocrit and plasma proteins and colloid osmotic pressure), lung permea­bility (non-invasive radionuclide method, 7-12,20,29), EVLW (with thermal/dye indicator diluti­on, 4,17,18,26,27), radio­graphic abnorma­lities sugge­stive for edema (28), pulmonary and systemic hemodyna­mics, gas exchan­ge and thus the so called composi­te semiquantitati­ve lung injury score (LIS, Appendix 1),

1) in 4 groups of 6 subjects each, of critically ill pa­tients with ARDS (defined according to American-European Consensus Conference criteria, Appendix I) and

2) in 4 patient groups at risk for (but yet without) ARDS: 4x6 pa­tients with multiple trau­ma and an injury severity score above 15 and non-vascular surgery (Appendix), 4x6 patients with sepsis according to the ACCP/SCCM consensus conference definitions (Appendix 1), 4x6 patients with major vascular surgery and 4x6 cardiac surge­ry involving cardiopulmonary bypass,

AND hypovolemia for which fluid infusion is clinic­ally requi­red, as defined by

A) hypo­tensi­on, ie systolic arterial blood pressure below 100 mm Hg (femoral arterial catheter in place) with or without inotropic treatment, or systolic arterial blood pressure between 100 and 110 mm Hg in the presence of significant inotropic support masking hypovolemia, and

B) reduced filling pressures, ie pulmonary capillary wedge pres­sure (PCWP) at or below 10 mm Hg in the presence of a pulmonary artery catheter, or a central venous pressure (CVP) at or below 8 mm Hg in the presence of a central venous catheter.

**Study de­sign.**

The studies proposed are prospective, randomized, non-blinded, observational stu­dies with pathophysiologic endpoints. Per study group (n=5), the hospital pharmacist will randomize among resuscitation fluids, according to a predefined list. Primary variables include the pulmonary microvascular protein permeability and EVLW. Secondary variables include lung mechanical and gas exchange varia­bles, plasma colloid osmotic pressure, course of the hematocrit, systemic and pulmonary hemodynamics and the chest radiograph. During ARDS, fluid loading with starch is hypothesized to give less rise to increased microvascular protein and fluid transport and pulmonary deterioration than loading with the other colloid and crystalloid fluids, the latter having no major differences. In patients at risk for ARDS (irrespective of underlying etiology), it is hypothezised that colloid fluids are equivalent and superior to crystalloids.

**Selection of patients.**

The patients will be recruited among consecutive admissions into the Medical and Surgical Intensive Care Units in predefined subgroups. Exclusi­on: age less than 18 and over 75 years of age, pregnancy, a preterminal illness (life expectancy of less than 24 h), known significant neurotrauma, and known anaphylactoid reactions to colloids. Patients will only be included after informed consent (of patient or closest relative) has been obtained and this will be recorded in the charts. Patients older than 70 years will be included since this age group is at particular risk for major surgery, severe illness needing intensive care and hypovolemic shock necessitating fluid resuscitation. Patients with ARDS will be recruited according to criteria of the American-European consensus conference, multiple trauma according to criteria of the Injury Severity Score (above 15) and sepsis according to the ACCP/SCCM consensus conference definitions (Appendix 1). Patients admitted for elective abdominal (aortic) and cardiac surgery (utilizing cardiopulmonary bypass in the latter) will also be recruited. Eligible patients will only be included when they already have or need, on clinical grounds and at the discretion of the treating physician, continuous arterial pressure monitoring and monitoring via a pulmonary artery or central venous catheter. Only patients on mechanical ventilation will be included.

**Materials and methods; protocol.**

The fluids under study are all registered and commonly used for resuscitation from hypovolemia. Hemohes (6% hydroethyl starch MW 200.000, substitution 0.45-0.55), Gelofusine (40 g/L), albumin (5%) and normal saline (0.9%) will be supplied by the sponsor (B. Braun Melsungen, Germany). For the non-invasive assessment of pulmonary microvascular permeabi­li­ty a new technique developed by our group will be used, the ^67^Ga-transferrin pulmonary leak index PLI, involving ^67^ Ga bound to circulating transferrin in vivo, and ^99m^Tc in bound to erythrocytes in vitro (7-12,29,30). The technique been described elsewhere. In short, 10 min after intravenous administration of 5 mg pyrophosphate (TechneScan, Mallinckrodt Medical, Petten), 10 mL of blood is obtained and equilibrated with 300 μCi (11 MBq) ^99m^Tc. Ten min later the blood is reinjected. In vivo labeling of transferrin occurs after injection of ^67^Ga-citrate (100 μCi, 4 MBq for the preinfusion study and 100 μCi, 4 MBq, for the postinfusion study). This is the only currently available non-invasive technique to measure pulmonary microvascular permea­bility and is available at only few places in the world. For the measure­ment of EVLW the thermal/dye thermodilution technique with help with the so called COLD^R^ machine (Pulsion, Munchen, Germany) will be used, yielding the gold standard for assessment of pulmonary edema (4,17,18,26,27). This measurement involves central venous injection of a dye and thermal bolus and concomitant registration of the dye dilution and thermal shift in the femoral artery, with help of a 5F catheter for pressure monitoring, equipped with a thermistor and fiberoptic. All other (hemodynamic and labora­tory) variables are stand­ard.

After recording demographics (including APACHE II score; Appendix I) and baseline measurements of the ^67^Ga-transferrin PLI (8x 5 mL blood samples in 0.5 h), the EVLW with the thermal/dye dilution technique, hemodynamics and blood samples for hematocrit/gas exchange parameters/protein levels/colloid osmotic pressure (5 mL), ventilator and chest radiograph variables, the fluids infusion regimens will be started. Fluids will be dosed on the basis of the response within predefined pressure limits, as measu­red by the pulmo­nary arterial catheter, ie fluid loading up to a PCWP of 15 mm Hg or a CVP of 13 mm Hg, by a standard fluid challenge protocol (see Appendix I), in 2 h. In this period, the amount of fluid loaded should not exceed 2 liters for colloid and 4 liters for crystalloid solutions. Attempts will be done to leave concomitant (particularly inotropic) treatment unchanged during the resuscitation period. The measurements will be repeated 2 h after discontinuation of the fluid loading (except for the radionuclide method and the chest radiograph), to evaluate the effect and extent of persistence of infused fluids in the circulation. Close surveillance of pulse oximetry will be used during fluid loading.

**Analysis.**

We feel a formal power analysis for these pathophysiolo­gical studies is inappropriate since the studies do not aim at evaluating a survival benefit of one fluid over the other. Statistical analysis will be done with help of non-parametric paired tests with respect to variables after fluid infusion versus baseline, in each of 5 groups. For the ARDS and for each ARDS at risk subgroup, fluids will be compared with help of non-parametric analysis of variance for repeated measures, with post hoc comparison by Mann-Whitney U tests, corrected for multiple comparisons with help of the Bonferroni-Holm statistic. An analysis will also be done for comparison of fluids, using the study population as a whole and with help of similar techniques. Non-linear and non-parame­tric regression analysis will be done to determine the main factors contri­buting to a change in the LIS after fluid infusion.

**Documentation and safety monitoring.**

All studies will be performed in accordance with good clinical practise and data will be recorded and stored both on paper and on computer disks.

Safety limits: infusion of fluids will be stopped and the patients excluded for further study and analysis if there is a (likely) anaphylactoid reaction with hypotensi­on, rashes etc. The fluid loading will be stopped if the pulse oxymetry O_2_ saturation falls below 87 % at an inspiratory O_2_ fraction of 60% or greater.

All adverse on therapy events ("AOTE's") will be recorded. Death and all major AOTE's should be reported to the study monitor within 24 hours. The severity of events will be graded as follows:

**Mild:** no treatment required, does not complicate the primary disease, suspected drug may or may not be stopped.

**Moderate:** Symptoms are marked, but involvement of vital organ systems is moderate. No loss of consciousness. No cardiovascular failure. Treatment or hospitalization required, or hospitalization prolonged by at least one day. Development of definite bioche­mical or structural changes could justify this classification.

**Severe:** An adverse drug event that is life-threatening or permanently disabling and required inpatient hospitalization. In addition, an adverse on therapy event with one of the following outcomes is always considered serious: death, congenital anomaly, can­cer, overdose.

The relation of AOTE's to the drugs under investigation is determined by the investigator as:

**Definite:** Follows a reasonable temporal sequence from administration of the drug, abates upon discontinuation of the drug. Is confirmed by reap­pearance of the reaction on repeated exposure (rechallenge).

**Probable:** A reaction that follows a reasonable temporal se­quence from administration of the drug; that follows a known or expected response pattern to the suspected drug; that abates by stopping or reducing dosage of the drug and that could not be reasonably explained by the known characteristics of the patient's clini­cal state.

**Possible:** A reaction that follows a reasonable temporal se­quence from administration of the drug; that follows a known or expected reponse pattern to the suspected drug; could have been produced by the patient's clinical state or by other modes of therapy administered to the patient.

**Remote:** A reaction that follows a reasonable temporal se­quence from administation of the drug; that does not follow a known respon­se pattern to the suspected drug and that could reasonably be explained by known characteristics of the patient's clinical state.

**Unknown:** Relationship for which no evaluation can be made.

**Unrelated:** A reaction for which sufficient information exists to indicate that etiology in unrelated to the study drug.

**Patient inconvenience.**

Apart from blood sampling, the radioactivity dose of ^99m^Tc (physical half live 6 hr) and of ^67^Ga (physical half live 78 h) used for the study is routine for measurement of the PLI (9) and is safe. The absorbed dose is 444 μSv for ^67^Ga and 94 μSv for ^99m^Tc per study, at a natural background of about 2400 μSv per year. Hence, the additional radiation dose is about 40 % of the yearly load, which is acceptable. Since inclusion criteria include arterial (5F fiberoptic, femoral artery) and pulmonary catheters in place, the thermal dye dilution technique can be used to evaluate the EVLW without any additional discomfort for the patients. The additional blood sampling for the study will not exceed 100 mL.

**Ethical considerations and informed consent.**

After protocol approval by the sponsor, the studies proposed in this programme will submitted to the scientific and ethical committees of the Institute of Cardiovascular Research at the VU (ICARVU) and the Academisch Ziekenhuis Vrije Universiteit, respectively, for approval. The study will be performed in accordance with the Declaration of Helsinki (see Appendix II). The Hospital Committee on Ethics will have to approve the protocol. Patients will only be included after informed consent from the patient or the closest relative has been ob­tained and this will be recorded in the patients' records (Appendix III). The sponsor will have free access to the (anony­mous) patient data, if requested.

**References.**

1. Thijs LG. Fluid therapy in septic shock. In: Sibbald WJ and Vincent J-L. Clinical trials for the treatment of sepsis. Update in Intensive Care and Emergency Medicine. Volume 19, Springer Verlag, Berlin, 1995, p 167-190

2. Traylor RJ, Pearl RG. Crystalloid versus colloid: all colloids are not created equal. Anesth Analg 1996;83:209-212

3. Schierhout G, Roberts I. Fluid resuscitation with colloid or crystalloid solutions in critically ill patients: a systematic review of randomised trials. Br Med J 1998;316:961-964

4. Schuller D, Mitchell JP, Calandrino FS, Schuster DP. Fluid balance during pulmonary edema. Is fluid gain a marker or a cause of poor outcome. Chest 1991;100:1068-1075

5. Sibbald WJ, Driedger AA, Wells GA, Myers ML, Lefcoe M. The short-term effects of increasing plasma colloid osmotic pressure in patients with noncardiac pulmonary edema. Surgery 1983;93:620-633

6. Groeneveld ABJ, Raijmakers PGHM. The ^67^Ga transferrin pulmonary leak index in patients at risk for the acute respiratory distress syndrome. Crit Care Med 1998;26:685-691

7. Raijmakers PGHM, Groeneveld ABJ, Schneider AJ, Teule GJJ, Lingen A van, Eysman L, Thijs LG. Transvascu­lar transport of ^67^Ga in the lungs after cardiopulmonary bypass surgery. Chest 1993;104:1825-1832

8. Raijmakers PGHM, Groeneveld ABJ, Rauwerda JA, Schneider AJ, Teule GJJ, Hack CE, Thijs LG. Transient increase in interleukin-8 and pulmonary microvascular permea­bility following aortic surgery. Am J Respir Crit Care Med 1995;151:698-705

9. Groeneveld ABJ. Radionuclide assessment of pulmonary microvascular permeability. Eur J Nucl Med 1997;24:449-461

10. Raijmakers PGHM, Groeneveld ABJ, Rauwerda JA, Teule GJJ, Hack CE. Acute lung injury after aortic surge­ry: the relation between lung and leg microvas­cular permeabi­lity to ^111^Indium-transferrin and circulating mediators. Thorax 1997:52:866-871

11. Groeneveld ABJ, Raijmakers PGHM, Teule GJJ, Thijs LG. The ^67^Gallium pulmonary leak index in assessing the seve­rity and course of the adult respiratory distress syndro­me. Crit Care Med. 1996;24:1467-1472

12. Raijmakers PGHM, Groeneveld ABJ, Teule GJJ, Thijs LG. The diagnostic value of the ^67^Gallium pulmonary leak index in pulmonary edema. J Nucl Med 1996;37:1316-1322

13. Erstad BL, Gales BJ, Rappaport WD. The use of albumin in clinical practice. Arch Intern Med 1991;151:901-911

14. Warren BB, Durieux ME. Hydroxyethyl starch: safe or not ? Anesth Analg 1997;84:206-212

15. Tanaka H, Dahms TE, Bell E, Naunheim KS, Baudendistel LJ. Effect of hydroxyethyl starch on alveolar flooding in acute lung injury in dogs. Am Rev Respir Dis 1993;148:852-859

16. Morisaki H, Bloos F, Keys J, Martin C, Neal A, Sibbald WJ. Compared with crystalloid, colloid therapy slows progression of extrapulmonary tissue injury in septic sheep. J Appl Physiol 1994;77:1507-1518

17. Gallagher JD, Moore RA, Kerns D, Jose AB, Botros SB, Flicker S, Naidech H, Clark DL. Effects of colloid or crystalloid administration on pulmonary extravascular water in the postoperative period after coronary artery bypass grafting. Anesth Analg 1985;64:753-758

18. Karanko MS, Klossner JA, Laaksonen VO. Restoration of volume by crystalloid versus colloid after coronary artery bypass: hemodynamics, lung water, oxygenation and outcome. Crit Care Med 1987;15:559-566

19. Boldt J, Heesen M, Müller M, Pabsdorf M, Hempelmann G. The effects of albumin versus hydroxyethyl starch solution on cardiorespiratory and circulatory variables in critically ill patients. Anesth Analg 1996;83:254-261

20. Zikria­ BA, King TC, Stan­ford J, Freeman HP. A biophysi­cal ap­proach to capillary permeabi­lity. Surgery 1989;105­:625-631

21. Vincent JL. Plugging the leaks ? New insights into synthetic col­loids. Crit Care Med 1991;19:316-318

22. Collins RE, Collins PW, Gutteridge CN, Kaul A, Newland AC, Williams DM, Webb AR. The effect of hydroxyethyl starch and other plasma volume substitues on endothelial cell activation; an in vitro study. Intens Care Med 1994;20:37-41

23. Boldt J, Mueller M, Heesen M, Neumann K, Hempelmann GG. Influence of different volume therapies and pentoxifylline infusion on circulating soluble adhesion molecules in critically ill patients. Crit Care Med 1996;24:385-391

24. Traber LD, Brazeal BA, Schmitz M, Toole J, Coffey J, Flynn JT, Traber DL. Pentafraction reduces the lung lymph response after endotoxin administration in the ovine model. Circ Shock 1992;36:93-103

25. Lum H, Siflinger-Birnboim A, Blumenstock F, Malik AB. Serum albumin decreases transendothelial permeability to macromolecules. Microvasc Res 12991;42:91-102

26. McLuckie A. The COLD system of haemodynamic monitoring. Intens Care World 1996;13:24-28

27. Godje O, Peyerl M, Seebauer T, Dewald O, Reichart B. Reproducibility of double indicator dilution measurements of in intrathoracic blood volume compartments, extravascular lung water, and liver function. Chest 1998;113:1070-1077

28. Halperin BD, Feeley TW, Mihm FG, Chiles C, Guthaner DF, Blank NE. Evaluation of the portable chest roentgenogram for quantifying extravascular lung water in critically ill adults. Chest 1985;88:649-654

29. Raijmakers PGHM, Groeneveld ABJ, De Groot MCM, Teule GJJ, Thijs LG. Delayed resolution of pulmonary oedema after cocaine/heroin abuse. Thorax 1994;49:1038-1040

30. Groeneveld ABJ, Raijmakers PGHM. The ^67^Ga transferrin pulmonary leak index during pneumonia and associated adult respiratory distress syndrome. Clin Sci 1997;93:463-470

**Appendix I.**

**Sepsis** (American College of Chest Physicians/Society of Critical Care Medicine):

Two or more of the following:

Abnormal body temperature (>38 °C, <36 °C)

Tachycardia (≥ 90/min)

Tachypnea (≥20/min) or P_a_CO_2_ <32 torr

Abnormal white blood cell counts (≤4, ≥12 x10^9^/L or >10% immature bands).

+ clinically evident focus of infection

**ARDS** (American-European Consensus Conference):

Oxygenation: P_a_O_2_/F_i_O_2_ <200 (regardless of PEEP)

Chest radiograph: bilateral infiltration seen on frontal chest radiograph

Pulmonary capillary wedge pressure: 18 mm Hg when measured or no clinical evidence of left atrial hypertension (lack of reaction to furosemide)

**Lung injury score**

**Chest radiograph**

No alveolar consolidation 0

Alveolar consolidation in 1 quadrant 1

Alveolar consolidation in 2 quadrant 2

Alveolar consolidation in 3 quadrant 3

Alveolar consolidation in 4 quadrant 4

**Hypoxemia score**

Arterial PO_2_/F_i_O_2_ 225-299 1

Arterial PO_2_/F_i_O_2_ 175-224 2

Arterial PO_2_/F_i_O_2_ 100-174 3

Arterial PO_2_/F_i_O_2_ 100 4

**Respiratory compliance, mL/cm H_2_O**

Compliance ≥80 0

Compliance 60-79 1

Compliance 40-59 2

Compliance 20-39 3

Compliance ≤19 4

**Positive end-expiratory pressure (PEEP, cm H_2_O)**

PEEP ≤5 0

PEEP 6-8 1

PEEP 9-11 2

PEEP 12-14 3

PEEP ≥15 4

According to Murray JF et al. An expanded definition of the adult respiratory distress syndrome. Am Rev Respir Dis 1988;138:720-723

F_i_O_2_= inspiratory O_2_ fraction. Score = sum/number of items.

**Fluid resuscitation protocol**:

**CVP, mmHg**

**Start Infusion During infusion After 10 min infusion**

CVP ≤8 200 ml/10 min Increase >5: stop Increase >5: stop

CVP <12 100 ml/10 min Increase 2-5: wait 10 min

Increase >2: stop

Increase ≤2: continue

CVP =12 50 ml/10 min Increase ≤2: continue

**PCWP, mmHg**

**Start Infusion During infusion After 10 min infusion**

PCWP ≤12 200 ml/10 min Increase >7: stop Increase >7: stop

PCWP <14 100 ml/10 min Increase 2-7: wait 10 min

Increase >3: stop

Increase ≤3: continue

PCWP =14 50 ml/10 min Increase ≤3: continue

According to Weil MH, Henning RJ. New concepts in the diagnosis and fluid treatment of circulatory shock. Anesth Analg 1979;58:124-132

**Appendix** **II. Radiology.**

1 - Chest radiography, just before and at 2 h after start of fluid loading. The first chest X-ray will, if possibly, be made in the morning concomitantly with the other patients' chest X-rays. Mark lower rib cage so that basal lung fields will be included on the chest X-ray. Standard exposure with focus-film distance of 1.3 m, multipulse, 99-105kV en 0.5 mAs without grid, on a mobylet 4 (Siemens). The chest X-rays will be made with help of regular lanex casettes with T-mat G film from Kodak. A sticker is applied on the cassette and the apllication form and Van den Berg is notified.

2 - Routing chest X ray. At the intensive care desk, in a separate locker 'research chest x rays', for 1 week; thereafter the exposures will be kept by Van den Berg.

3 - Interpretation. By the radiologist on call for the intensive care unit; an extra document will be prepared and attached to the exposure. Later the chest X-rays will be judged by two independent radiologists. A consensus will be reached according to predefined criteria (see below).

4 - Digitalisation. The exposures will be scannded and density measurements (relative to fixed points) will be performed.

6 - Planning: 120 patients in 3 years (2x2 exposures/week).

7 - The results of the chest X-ray will be compared with the other measurements of the study and papers will be prepared that will address the value of the chest X-ray as compared to direct measurements of (changes) in extravascular lung water (edema).

Criteria chest X-ray.

Phase

I: Peribronchial cuffing en vafue vessel contours

II: Kerley-B en kerley-A

III: Perihilar haze

IV: Thickening of fissures/pleural fluid

V: Alveolar edema

Phase V location alveolar edema (left/right)

A perihilar

B central

C peripheral

a upper fields

b middle fields

c lower fields

**Appendix** **III. Declaration of Helsinki.**

Recommendations guiding medical doctors in biomedical research involving human subjects. Adopted by the 18^th^ World Medical Assembly, Helsinki, Finland, 1964 and as revised by the 29^th^ World Medical Assembly, Tokyo, Japan, 1975 and by the 35^th^ World Medical Assembly, Venice, Italy, 1983.

Introduction.

It is the mission of the medical doctor to safeguard the health of the people. His or her knowledge and conscience are dedicated to the fulfillment of this mission. The Declaration Of Geneva of the World Medical Association binds the physician with the words. "The health of my patient will be my first consideration" and the International Code of Medical Ethics declares that "A physician shall act only in the patient's interest when providing medical care which might have the effect of weakening the physical and mental condition of the patient". The purpose of biomedical research involving human subjects must be to improve diagnostic, therapeutic and prophylactic procedures and the understanding of the etiology and pathogenesis of disease. In current medical practice most diagnostic, therapeutic or prophylactic procedures involve hazards. This applies especially to biomedical research. Medical progress is based on research which ultimately must rest in part on experimentation involving human subjects. In the field of biomedical research a fundamental distinction must be recognized between medical research in which the aim is essentially diagnostic or therapeutic for a patients and medical research, the essential object of which is purely scientific and without implying direct diagnostic or therapeutic value to the person subjected to research. Special caution must be exercised in the conduct of research which might affect the environment and the welfare of animals used for research must be respected. Because it is essential that the results of laboratory experiments be applied to human beings to further scientific knowledge and to help suffering humanity. The World Medical Association has prepared the following recommendations as a guide to every physician in biomedical research involving human subjects. They should be kept under review in the future. It must be stressed that the standards as drafted are only a guide to physicians all over the world. Physicians are not relieved from criminal, civil and ethical responsibilities under the laws of their own countries.

I. Basic principles.

1. Biomedical research involving subjects must conform to generally accepted scientific principles and should be based on adequately performed laboratory and animal experimentation and on a thorough knowledge of the scientific literature.

2. The design and performance of each experimental procedure involving human subjects should be clearly formulated in an experimental protocol which should be transmitted to a specially appointed independent committee for consideration, comment and guidance.

3. Biomedical research involving human subjects should be conducted only by scientifically qualified persons and under the supervision of a clinically competent medical person. The responsibility for the human subject must always rest with a medically qualified person and never rest in the subject of the research, even though the subject has given his or her consent.

4. Biomedical research involving human subjects cannot legitimately be carried out unless the importance of the objective is in proportion to the inherent risk to the sub­ject.

5. Every biomedical research project involving human subjects should be preceded by careful assessment of predictable risks in comparison with foreseeable benefits to the subject or to others. Concern for the interest of the subject must always prevail over the interests of science and society.

6. The right of the research subject to safeguard his or her integrity must always be respected. Every precaution should be taken to respect the privacy of the subject and to minimize the impact of the study on the subject's physical and mental integrity and on the personality of the subject.

7. Physicians should abstain from engaging in research projects involving human subjects unless they are satisfied that the hazards involved are believed to be predictable. Physicians should cease any investigation if the hazards are found to outweigh the potential benefits.

8. In publication of the results of his or her research, the physician is obliged to preserve the accuracy of the results. Reports of experimentation not in accordance with the prin­ciples laid down in this Declaration should not be accepted for publication.

9. In any research on human beings, each potential subject must be adequately informed of the aims, methods, anticipated benefits and potential hazards of the study and the discomfort it may entail. He or she should be informed that he or she is at liberty to abstain from participation in the study and that he or she is free to withdraw his or her consent to participation at any time. The physician should than obtain the subject's freely-given informed consent, preferable in writing.

10. When obtaining informed consent for the research project the physician should be particularly cautious if the subject is in a dependent relationship to him or her or may consent under duress. In that case the informed consent should be obtained by a physician who is not engaged in the investigation and who is completely independent of this of­ficial relationship.

11. In case of legal incompetence, informed consent should be obtained from the legal guardian in accordance with national legislation. Where physical or mental incapacity makes it impossible to obtain informed consent, or when the subject is a minor, permission from the responsible relative replaces that of the subject in accordance with national legislation. Whenever the minor child is in fact able to give a consent, the minor's consent must be obtained in addition to the con­sent of the minor's legal guardian.

12. The research protocol should always contain a statement of the ethical considerations involved and should indicate that the principles enunciated in the present Declaration are complied with.

II. Medical research combined with professional care (clinical research).

1. In the treatment of the sick person, the physician must be free to use a new diagnostic and therapeutic measure, if in his or her judgement if offers hope of saving life, reestablishing health or alleviating suffering.

2. The potential benefits, hazards and discomfort of a new method should be weighed against the advantages of the best current diagnostic and therapeutic methods.

3. In any medical study, every patient -including those of a control group, if any- should be assured of the best proven diagnostic and therapeutic method.

4. The refusal of the patient to participate in a study must never interfere with the physician-patient relationship.

5. If the physician considers it essential not to obtain informed consent, the specific reasons for this proposal should be stated in the experimental protocol for transmission to the independent committee (I.2).

6. The physician can combine medical research with profes­sional care, the objective being the acquisition of new medical knowledge, only to the extent that medical research is justified by its potential diagnostic or therapeutic value for the patient.

III. Nontherapeutic biomedical research involving human sub­jects (nonclinical biomedical research).

1. In the purely scientific application of medical research carried out on a human being, it is the duty of the physician to remain the protector of the life and health of that person on whom biomedical research is being carried out.

2. The subjects should be volunteers - either healthy persons or patients for whom the experimental design is not related to the patient's illness.

3. The investigator or the investigating team should discon­tinue the research if in his/her or their judgement it may, if continued, be harmful to the individual.

4. In research on man, the interest of science and society should never take precedence over considerations related to the well-being of the subject.
